# MRI pelvimetry-based evaluation of surgical difficulty in laparoscopic total mesorectal excision after neoadjuvant chemoradiation for male rectal cancer

**DOI:** 10.1007/s00595-020-02211-3

**Published:** 2021-01-09

**Authors:** Jianhua Chen, Yanwu Sun, Pan Chi, Bin Sun

**Affiliations:** 1 Department of Radiology, Fujian Medical University Union Hospital, 29 Xinquan Road, Fuzhou, 350001 Fujian People’s Republic of China; 2 Department of Colorectal Surgery, Fujian Medical University Union Hospital, Fuzhou, Fujian People’s Republic of China

**Keywords:** Rectal cancer, Chemoradiotherapy, Laparoscopic surgery, Pelvimetry, Magnetic resonance

## Abstract

**Purpose:**

Laparoscopic total mesorectal excision (LaTME) is technically demanding in rectal cancer after neoadjuvant chemoradiotherapy (NCRT). This study aimed to predict the surgical difficulty of LaTME after NCRT based on pelvimetric parameters.

**Methods:**

This study enrolled 147 patients who underwent LaTME after NCRT. The surgical difficulty was graded as high or low according to the operative time, estimated blood loss, conversion to open surgery, postoperative hospital stay, and postoperative complications. Pelvimetry parameters were collected based on preoperative MRI. A logistic regression analysis was performed to identify predictors of high surgical difficulty, and a nomogram was developed.

**Results:**

Totally, 18 (12.2%) patients were graded as high surgical difficulty. High surgical difficulty was correlated with a shorter interspinous distance (*P* = 0.014), a small angle *α* and *γ* (*P* = 0.008, *P* = 0.008, respectively), and a larger mesorectal area and mesorectal fat area (*P* = 0.041, *P* = 0.046, respectively). Tumor distance from the anal verge (OR = 0.619, *P* = 0.024), tumor diameter (OR = 3.747, *P* = 0.004), interspinous distance (OR = 0.127, *P* = 0.007), and angle *α* (OR = 0.821, *P* = 0.039) were independent predictors of high surgical difficulty. A predictive nomogram was developed with a C-index of 0.867.

**Conclusion:**

A shorter tumor distance from the anal verge, larger tumor diameter, shorter interspinous distance, and smaller angle *α* could help to predict high surgical difficulty of LaTME in male LARC patients after NCRT.

**Supplementary Information:**

The online version contains supplementary material available at 10.1007/s00595-020-02211-3.

## Introduction

Surgery is the cornerstone of treatment for rectal cancer [1]. Several randomized controlled trials have ascertained comparable oncological outcomes of laparoscopic rectal cancer surgery in comparison to open surgery, along with the short-term advantages in terms of postoperative pain, bowel function, and postoperative hospitalization [2–5]. Despite the increased application of laparoscopic procedures, laparoscopic total mesorectal excision (LaTME) for mid/low rectal cancers can be technically demanding, particularly in obese male patients with a narrow pelvis. Currently, neoadjuvant chemoradiotherapy (NCRT) followed by total mesorectal excision (TME) is accepted as the standard treatment for patients with locally advanced rectal cancer (LARC) [6, 7]. However, tissue inflammation, edema, or fibrosis following NCRT can impede vision and hamper dissection maneuvers, thereby adding to the surgical difficulty of laparoscopic surgery for rectal cancer after NCRT. Considering the technical and ergonomic advantages, robotic TME might help to overcome the limitations of LaTME in the confines of the pelvis [8]. Additionally, a new down-to-up approach to rectal cancer surgery, transanal TME (TaTME), appears to be an alternative surgical option for rectal cancers, especially in individuals with a narrow pelvis [9]. Therefore, the preoperative evaluation of surgical difficulty could help to plan the optimal surgical approach.

Besides surgical skills, there are several well-established factors associated with the increased surgical difficulty of LaTME, including male sex, high body mass index (BMI), prior abdominal surgery, a low-lying tumor, and advanced tumor stage [10, 11]. The pelvic anatomy can also influence the operative difficulties of LaTME, including—but not limited to—a prominent sacral promontory, an acutely curved sacrum, or a narrow pelvic outlet. Recently, magnetic resonance imaging (MRI)-based pelvimetry, including the pelvic dimensions and angles, has been proposed as a useful tool for evaluating the surgical difficulties of LaTME [12–14]. As expected, LaTME in a narrow pelvis can be more difficult to perform due to radiation-induced tissue inflammation, edema, or fibrosis. Besides, male patients with rectal cancer usually represent more challenging cases to surgeons, given that the female pelvis is generally more accessible than the male pelvis during pelvic surgery. Thus, performing LaTME in male patients after NCRT is expected to be more technically challenging.

However, the surgical difficulties of LaTME in male rectal cancer patients following NCRT have not been robustly explored [13, 15]. To address the gap in the literature, we aimed to investigate the clinical and pelvimetric factors that predict surgical difficulties of LaTME after NCRT and to develop a predictive nomogram to assist in the selection of the optimal surgical approach for mid/low rectal cancer after NCRT.

## Patients and methods

### Patients

Consecutive male rectal cancer patients who underwent NCRT and laparoscopic surgery in our institution between 2015 and 2016 were identified for this study. The patient inclusion criteria were as follows: (1) tumor within 10 cm from the anal verge; (2) pathologically proven adenocarcinoma; (3) clinically staged as T3/4 and/or N+ disease; (4) patient underwent NCRT followed by LaTME, and (5) sufficient preoperative MRI data. Patients who underwent abdominoperineal resection (APR), Hartmann’s procedure, robotic surgery, and TaTME, or other types of surgery (e.g., emergency or palliative surgery, pelvic exenteration, para-aortic, or lateral pelvic lymphadenectomy) were excluded. This study was approved by the institutional review board of our hospital.

### Treatment

All patients underwent long-course NCRT before radical surgery. Preoperative radiotherapy was delivered via three-dimensional conformal radiation therapy (3D-CRT) or intensity-modulated radiation therapy (IMRT) at a dose of 45–50.4 Gy (1.8–2.0 Gy/day × 25–28 fractions for 5–6 weeks). Chemotherapy was administered concurrently with radiation using one of the following chemotherapeutic regimens, including capecitabine plus oxaliplatin (CapeOX) or 5-fluorouracil/folinic acid plus oxaliplatin (FOLFOX). At approximately 8 weeks after the completion of radiation therapy, surgery was planned by a highly experienced surgical team. Laparoscopic surgery for mid/low rectal cancer consisted of low anterior resection (LAR), ultra-low anterior resection (ULAR), and intersphincteric resection (ISR). LaTME was performed according to the principle of TME, and high ligation of the inferior mesenteric artery (IMA) was routinely performed [16]. When needed, partial or complete mobilization of the splenic flexure was performed to ensure tension-free colorectal or coloanal anastomosis. Pelvic dissection was performed from the sacral promontory down to the pelvic floor. Then, the rectum was transected and reconstructed by colorectal or coloanal anastomosis. Diverting ileostomy was fashioned to protect the anastomotic site when the anastomotic height was ≤ 5 cm from the anal verge, or if the patient had a poor nutritional status or diabetes. Conversion to open surgery was needed when it was impossible to complete the procedure laparoscopically.

### Definition of surgical difficulty

The surgical difficulty was defined using both intraoperative and postoperative parameters; the method was modified from that of Escal et al. [12]. The postoperative complications were graded according to the Clavien–Dindo classification [17]. Grade II complications were defined as any event that required medication, blood transfusion, or total parenteral nutrition. Grade III complications were defined as any event that required surgical, endoscopic, or radiological intervention. Complication was defined as Grade II or III surgical complications, such as anastomotic leakage, anastomotic bleeding, peritoneal bleeding, surgical site infections, and bowel obstruction. The surgical difficulty was scored as follows: operative time > 300 min (score 3), blood loss > 200 ml (score 1), conversion to open surgery (score 3), Clavien–Dindo grades II and III postoperative complications (score 1), and the use of transanal dissection (score 2), and postoperative hospital stay > 7 days (score 2). Based on the total score for surgical difficulty, the patients were classified into the low (score 0–2) and high (score ≥ 3) surgical difficulty groups.

### MRI-based pelvimetry

**Fig. 1:**
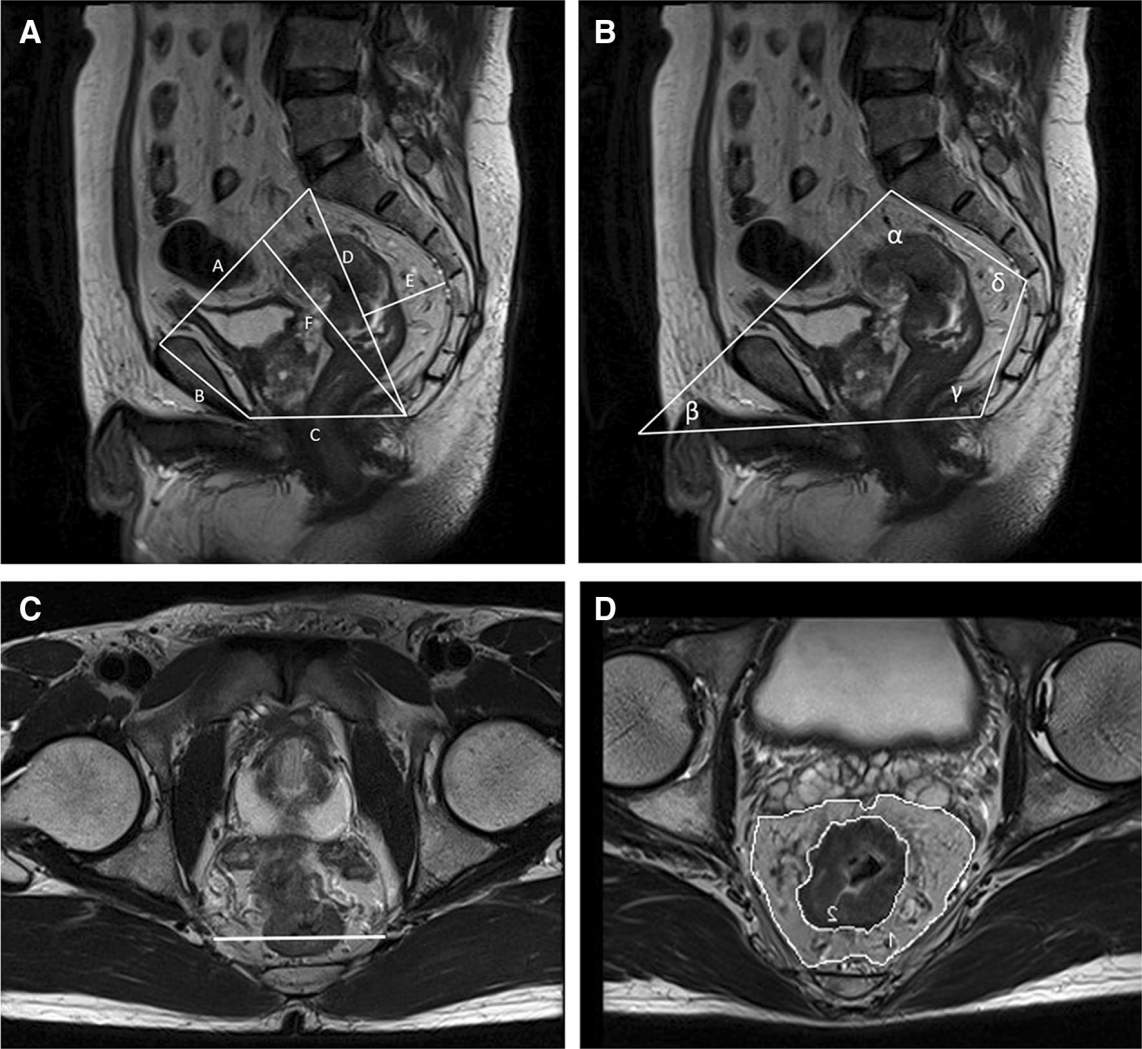
Images showing the MRI-based pelvimetric parameters. **a** Sagittal MRI showing the pelvic inlet (A), pubic tubercle height (B), pelvic outlet length (C), sacral length (D), sacral depth (E), and pelvic depth (F). **b** Sagittal MRI illustrating the pelvic angles (*α*, *β*, *γ*, and *δ*). **c** Axial MRI showing the interspinous distance. **d** Axial MRI showing the manual tracing of the circumference of the rectum (1), which represented the rectal area; axial MRI showing the manual tracing of the circumference of the mesorectum (2), which represented the mesorectal area

Pelvic MRI examinations were performed using a 3.0 T Siemens Prisma human MRI scanner (MAGNETOM Trio, Siemens Healthcare, Erlangen, Germany) or a 3.0 T GE MR Scanner (Discovery 750 W system, General Electric Healthcare System, Milwaukee, WI, USA). Using an Advantage Workstation 4.6 (GE Medical Systems), all pelvic MR images were retrospectively reviewed by one radiologist (CJH) who was blinded to the patients’ clinicopathological information. Pelvimetry dimensions and angles were obtained using mid-sagittal and axial MRI scans, as described previously [18], which was demonstrated in Fig. 1. Mesorectal and rectal contours were traced manually. The detailed definitions of the pelvimetric parameters are listed in Supplementary Table 1.

### Statistical analysis

All statistical analyses were performed using SPSS version 25 (IBM SPSS INC. Chicago, IL, USA). Data were described as the number and percentage or mean ± standard deviation and were assessed using the Chi-squared test or Student’s *t *test, when appropriate. Risk factors for the surgical difficulty of LaTME were determined with a logistic regression model. Then, based on the risk factors, a predictive nomogram was constructed using R version 3.5.1 (http://www.r-project.org/). The nomogram was internally validated by bootstrapping. The discriminative ability of the nomogram was evaluated by the concordance index (C-index). Calibration of the nomogram was performed by comparing the nomogram-predicted probability with the observed probability after bias correction. *P* values of < 0.05 were considered to indicate statistical significance.

## Results

### Patient characteristics

A total of 147 patients were eligible for inclusion in the analysis. The mean age was 56.4 ± 11.6 years, and the mean BMI was 23.1 ± 2.9 kg/m^2^. The tumor distance from the anal verge was 6.8 ± 2.0 cm. In total, 10 patients had a history of previous abdominal surgery. The time from the completion of radiation to surgery was 9.3 ± 4.3 weeks. The baseline characteristic parameters of patients are summarized in Table 1.

**Table 1:** Baseline characteristics of LARC patients following NCRT and LaTME

Characteristics	Values
Age (years)	56.4 ± 11.6
BMI (kg/m^2^)	23.1 ± 2.9
Distance from the anal verge (cm)	6.8 ± 2.0
Tumor diameter (cm)	2.0 ± 1.0
Prior abdominal surgery	10 (6.8%)
Time interval from completion of radiation to surgery (week)	9.3 ± 4.3
Surgical procedure	
LAR	52 (35.4%)
ULAR	77 (52.4%)
ISR	18 (12.2%)
Operative time (min)	227.5 ± 63.2
Estimated blood loss (ml)	70.9 ± 72.5
Conversion to open procedure	3 (2%)
Use of transanal dissection	3 (2%)
Postoperative hospital stay (days)	7.7 ± 3.8
Postoperative complications	23 (15.6%)
Pathological TNM stage	
0	30 (20.4%)
I	36 (24.5%)
II	32 (21.8%)
III	49 (33.3%)
Lymph node harvested	13.8 ± 7.2

Data are described as the number (percentage) or as the median ± standard deviation

*LARC* locally advanced rectal cancer, *NCRT* neoadjuvant chemoradiotherapy, *LaTME* laparoscopic total mesorectal excision, *BMI* body mass index, *LAR* low anterior resection, *ULAR* ultra-low anterior resection, *ISR* intersphincteric resection, *TNM* tumor node metastasis

### Surgical outcomes

Regarding the surgical procedure, LAR was performed in 52 (35.4%) patients, ULAR was performed in 77 (52.4%) patients, and ISR was performed in 18 (12.2%) patients. The operative time was 227.5 ± 63.2 min, and the estimated blood loss was 70.9 ± 72.5 ml. A total of 3 (2%) patients experienced conversion to open surgery, and 3 (2%) patients required transanal dissection. The postoperative hospital stay was 7.7 ± 3.8 days, and postoperative complications were seen in 23 (15.6%) patients. According to the grade of surgical difficulty, patients were divided into the low (*n* = 129) and high (*n* = 18) surgical difficulty groups.

### Pelvimetry parameters

As shown in Table 2, the mean pelvic inlet was 11.3 ± 0.9 cm, the mean pubic tubercle height was 5.2 ± 0.3 cm, and the mean pelvic outlet length was 7.9 ± 0.6 cm. The mean sacral length was 12.3 ± 1.1 cm, the mean sacral depth was 3.8 ± 0.4 cm, and the mean pelvic depth was 10.9 ± 0.8 cm. The mean interspinous distance was 8.7 ± 0.8 cm. The mean angle of angles *α*, *β*, *γ*, and *δ* was 87.1° ± 8.5°, 45.0° ± 7.4°, 118.2° ± 9.7°, and 109.2° ± 10.0°, respectively. The mean mesorectal area was 26.9 ± 5.9 cm^2^, the mean rectal area was 7.7 ± 3.2 cm^2^, and the mean mesorectal fat area was 19.1 ± 5.3 cm^2^. As demonstrated in Table 2, high surgical difficulty was correlated with a shorter interspinous distance (*P* = 0.014), as well as with a small angle *α* and *γ* (*P* = 0.008, *P* = 0.008, respectively). A larger mesorectal area and mesorectal fat area) was correlated with high surgical difficulty (*P* = 0.041, *P* = 0.046, respectively).

**Table 2:** Pelvimetry parameters in LARC patients following NCRT

Parameters	Total (*n* = 147)	Surgical difficulty		*P* value
		Low (*n* = 129)	High (*n* = 18)	
Pelvic inlet length (cm)	11.3 ± 0.9	11.3 ± 1.0	11.5 ± 0.8	0.346
Pubic tubercle height (cm)	5.2 ± 0.3	5.2 ± 0.3	5.2 ± 0.4	0.848
Pelvic outlet length (cm)	7.9 ± 0.6	7.8 ± 0.6	8.0 ± 0.6	0.300
Sacral length (cm)	12.3 ± 1.1	12.3 ± 1.2	12.3 ± 0.9	0.849
Sacral depth (cm)	3.8 ± 0.4	3.8 ± 0.5	3.7 ± 0.4	0.403
Pelvic depth (cm)	10.9 ± 0.8	10.9 ± 0.8	11.2 ± 0.6	0.167
Interspinous distance (cm)	8.7 ± 0.8	8.7 ± 0.8	8.2 ± 0.4	0.014
Angle *α* (°)	87.1 ± 8.5	87.8 ± 8.3	82.2 ± 8.4	0.008
Angle *β* (°)	45.0 ± 7.4	44.6 ± 7.4	48.0 ± 7.1	0.072
Angle *γ* (°)	118.2 ± 9.7	117.4 ± 9.6	123.9 ± 8.1	0.008
Angle *δ* (°)	109.2 ± 10.0	109.7 ± 10.1	106.0 ± 8.5	0.140
Mesorectal area (cm^2^)	26.9 ± 5.9	26.5 ± 6.0	29.6 ± 4.8	0.041
Rectal area (cm^2^)	7.7 ± 3.2	7.6 ± 3.3	8.1 ± 3.0	0.615
Mesorectal fat area (cm^2^)	19.1 ± 5.3	18.8 ± 5.4	21.5 ± 3.4	0.046

Data are expressed as the median ± standard deviation

*LARC* locally advanced rectal cancer, *NCRT* neoadjuvant chemoradiotherapy

### Predictors of high surgical difficulty in LaTME after NCRT

Univariate analysis demonstrated that older age (*P* = 0.041), higher BMI (*P* = 0.001), shorter tumor distance from the anal verge (*P* = 0.021), longer tumor diameter (*P* < 0.001), shorter interspinous distance (*P* = 0.016), smaller angle α (*P* = 0.010), larger angle *δ* (*P* = 0.010), larger mesorectal area (*P* = 0.043), and larger mesorectal fat area (*P* = 0.049) were significantly correlated with high surgical difficulty in patients undergoing LaTME after NCRT, as demonstrated in Table 3. No association was observed between surgical difficulty and previous abdominal surgery, or the time interval from the completion of radiation to surgery (*P* = 0.093, *P* = 0.624, respectively). Other pelvimetric parameters, such as pubic tubercle height, pelvic outlet length, sacral length, sacral depth, pelvic depth, rectal area, angle *β*, and angle *ε* were not significantly associated with the surgical difficulty of LaTME after NCRT (all *P* > 0.05). Multivariate analysis revealed that the tumor distance from the anal verge (OR = 0.619, 95% CI 0.409–0.938, *P* = 0.024), tumor diameter (OR = 3.747, 95% CI 1.538–9.129, *P* = 0.004), and interspinous distance (OR = 0.127, 95% CI 0.028–0.564, *P* = 0.007), and angle *α* (OR = 0.821, 95%CI 0.681–0.990, *P* = 0.039) independently predicted high surgical difficulty in LaTME after NCRT, as shown in Table 3.

**Table 3:** Logistic regression analysis of predictors of high surgical difficulty in LaTME for LARC following NCRT

Factors	Univariate analysis		Multivariate analysis	
	OR (95% CI)	*P* value	OR (95% CI)	*P* value
Age	0.957 (0.918–0.998)	0.041	0.935 (0.872–1.003)	0.062
BMI	1.416 (1.160–1.728)	0.001	1.333 (0.999–1.779)	0.051
Distance from the anal verge	0.719 (0.543–0.952)	0.021	0.619 (0.409–0.938)	0.024
Previous abdominal surgery	3.486 (0.814–14.934)	0.093		
Tumor diameter	2.975 (1.761–5.028)	< 0.001	3.747 (1.538–9.129)	0.004
Time interval from completion of radiation to surgery	0.917 (0.650–1.295)	0.624		
Pelvic inlet length	1.265 (0.777–2.059)	0.345		
Pubic tubercle height	1.142 (0.297–4.399)	0.847		
Pelvic outlet length	1.443 (0.722–2.886)	0.299		
Sacral length	0.959 (0.629–1.464)	0.848		
Sacral depth	0.651 (0.238–1.775)	0.401		
Pelvic depth	1.499 (0.842–2.669)	0.169		
Interspinous distance	0.421 (0.208–0.852)	0.016	0.127 (0.028–0.564)	0.007
Angle *α*	0.917 (0.858–0.979)	0.010	0.821 (0.681–0.990)	0.039
Angle *β*	1.070 (0.993–1.153)	0.075		
Angle *γ*	1.082 (1.019–1.149)	0.010	0.938 (0.807–1.090)	0.404
Angle *δ*	0.960 (0.910–1.014)	0.142		
Mesorectal area	1.093 (1.003–1.191)	0.043	0.833 (0.598–1.161)	0.281
Rectal area	1.038 (0.899–1.197)	0.612		
Mesorectal fat area	1.103 (1.001–1.215)	0.049	1.469 (0.978–2.206)	0.064

*LaTME* Laparoscopic total mesorectal excision, *LARC* locally advanced rectal cancer, *NCRT* neoadjuvant chemoradiotherapy, *OR* odds ratio, *CI* confidence interval, *BMI* body mass index

### A nomogram predicting high surgical difficulty in LaTME after NCRT

**Fig. 2:**
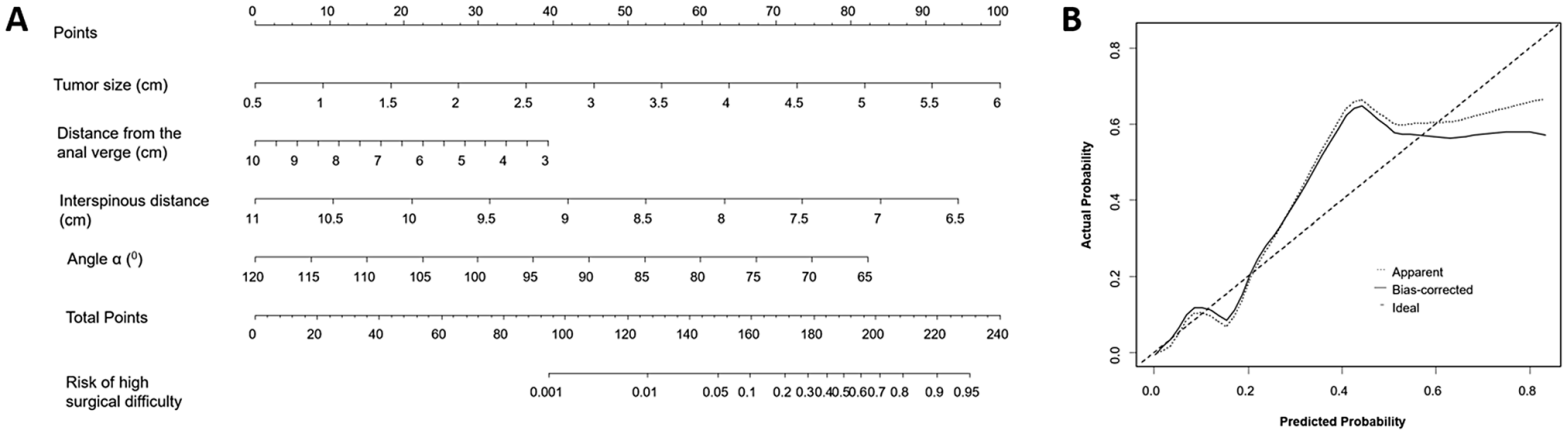
A nomogram for predicting the probability of experiencing high surgical difficulty in LaTME for LARC following NCRT. **a** A nomogram for predicting high surgical difficulty in LaTME for LARC after NCRT. **b** Calibration curves for the nomogram with internal validation. *LaTME* Laparoscopic total mesorectal excision, *LARC* locally advanced rectal cancer, *NCRT* neoadjuvant chemoradiotherapy

Based on these results, we developed a predictive nomogram for high surgical difficulty in LaTME after NCRT, as depicted in Fig. 2a. A higher total score was associated with a higher probability of high surgical difficulty. The C-index of the nomogram was 0.867 (95% CI 0.821–0.913). On internal validation, the calibration curve showed a similarity between the predicted and actual probability of high degree of surgical difficulty in LaTME after NCRT (Fig. 2b).

## Discussion

Currently, studies focused on the surgical difficulty of LaTME after NCRT for male rectal cancer patients are limited [13, 15]. The present study demonstrated that higher BMI, shorter tumor distance from the anal verge, larger tumor diameter, shorter interspinous distance, and smaller angle *α* could help predict the surgical difficulty of LaTME after NCRT. We then constructed a nomogram predicting the surgical difficulty of LaTME after NCRT, which may be helpful when selecting the surgical approach preoperatively.

BMI, an easily obtainable parameter of obesity, is also useful in predicting surgical difficulty[10]. We found that higher BMI values were associated with higher degree of surgical difficulty when performing LaTME in male patients following NCRT, which was consistent with previous findings. A larger tumor diameter usually indicates a larger tumor volume, which may restrict the pelvic working space, and thus increases the surgical difficulty of LaTME [19]. The pelvic space becomes narrower as rectal cancers approach closer to the anal verge when performing rectal dissection, transection, and anastomosis; thus, the surgical difficulty may increase as well [10]. Consistent with previous studies, the present study demonstrated that a larger tumor diameter and shorter tumor distance from the anal verge were independent predictors of the surgical difficulty of LaTME after NCRT.

Tumor downsizing may reduce the surgical difficulty of rectal cancer surgery. In our daily clinical practice, we have found that tumor downsizing in good responders to NCRT may facilitate surgical dissection. In the present study, we found that the tumor diameter independently predicted high surgical difficulty in laparoscopic TME in rectal cancer surgery (univariate *P* < 0.001, multivariate *P* = 0.004). While NCRT could induce tumor downsizing and downstaging, dissection of the mesorectum is often hindered by edema, mist, and exudates induced by NCRT, and thus adds to the surgical difficulty. Several surrogate markers have been utilized to estimate the surgical difficulty of TME, including the operative time, blood loss, conversion, circumferential resection margin (CRM) status, and postoperative complications [11, 20]. Considering that impaired surgical quality and an eventful postoperative course might compromise the oncological outcome and survival [21], we herein applied both intraoperative and postoperative parameters to better define surgical difficulty by modifying the definition previously proposed by Escal et al. [12].

Indeed, surgical expertise is one of the most important factors influencing the surgical difficulty of LaTME. In our study, surgeries were performed by a group of highly experienced surgeons. Among these cases, LaTME after NCRT was performed with a very low conversion rate (2%) and a low incidence of postoperative morbidity (15.6%); in 12.2% of the cases, LaTME after NCRT was considered to be associated with a high degree of surgical difficulty, which was similar to the rate reported by Escal et al. (12.8%) [12].

In general, male patients have a narrower and deeper pelvis than female patients, which may result in more challenging LaTME [22]. Pelvic anatomical factors, such as a narrow pelvis, a prominent sacral promontory, an acutely curved sacrum, and a shallow sacral angle represent anatomical bottlenecks of the pelvis and add to the surgical difficulty of LaTME for rectal cancer [15, 23]. In addition, these limitations cannot be completely overcome by surgical expertise. To date, there is increasing interest in MR-based pelvimetry to predict the surgical difficulty of LaTME [10, 11, 20]. However, the optimal pelvimetric parameters influencing surgical difficulty remain inconsistent in the literature. In this study, we used 14 pelvimetric parameters based on MRI, including 7 dimensions, 4 angles, and 3 areas of the pelvis. The univariate analyses demonstrated that shorter interspinous distance, smaller angle *α*, larger angle *δ*, larger mesorectal area, and larger mesorectal fat area were associated with high surgical difficulty in LaTME after NCRT. The multivariate analysis demonstrated that a shorter interspinous distance and smaller angle *α* were independently associated with a high degree of surgical difficulty, which was in good accordance with previous findings [12, 14]. A smaller angle *α* may limit the maneuverable space, and make for unsatisfying counter traction turns, thereby increasing the surgical difficulty of LaTME. Not surprisingly, our study also found that a shorter tumor distance from the anal verge was independently associated with high surgical difficulty of LaTME following NCRT. In addition, a larger tumor within the bony pelvis could increase the operative difficulty [24]. Similarly, our results reaffirmed that a larger tumor diameter was an independent predictor of high surgical difficulty in LaTME following NCRT.

Great efforts have been devoted to building scoring systems that predict the surgical difficulty of LaTME for rectal cancer [12, 14]. By incorporating both clinical and pelvimetric parameters, the present study developed a nomogram predicting cases in which LaTME after NCRT would be associated with high surgical difficulty; this nomogram showed good discriminative power. Using this nomogram, early surgical trainees can select appropriate cases to minimize adverse outcomes and reduce the impact of inexperience. Besides, patients could be informed of surgical difficulty as well as perioperative risks and complications. Currently, robotic rectal cancer surgery is gaining acceptance due to several advantages over laparoscopic surgery [25]. TaTME is a promising technique that could overcome the limitations of LaTME, especially in obese patients [26]. Our nomogram might assist in the preoperative selection of an appropriate surgical approach for LARC patients after NCRT (e.g., open, laparoscopic, robotic, or transanal).

Robotic TME might help to overcome the limitations of LaTME in the confines of the pelvis. As reported previously [8], high BMI, use of NCRT, and lower tumor levels were significantly associated with a longer operation time, which was in line with our findings. Different from our results, pelvimetric parameters were not associated with a longer operation time in patients undergoing robotic TME. One potential explanation could be the ergonomic advantages and improved dexterity of robotic TME. TaTME, which is TME with a down-to-up approach, is a promising alternative for rectal cancers with a narrow pelvis. Ferko et al. [27] found a correlation between TME quality and pelvimetric parameters, and suggested that it could be used as a tool for selecting candidates for TaTME.

The present study was associated with several limitations. First, this study was a monocentric study based on a retrospective analysis. Second, the sample size was relatively small. Third, higher surgical difficulty may potentially affect the quality of TME or pathological CRM and thus impairs the oncological outcome and survival [21]. Since the present study aimed to identify predictors of surgical difficulty of LaTME in male patients after NCRT, we did not evaluate oncological factors, which could be a limitation of our study. Fourth, our predictive nomogram required further validation in other independent patient cohorts. Despite these limitations, the present study might add to the understanding of the predictive value of MRI-based pelvimetry when estimating the surgical difficulty of LaTME in male LARC patients after NCRT.

## Conclusion

Our study demonstrated that higher BMI, shorter tumor distance from the anal verge, larger tumor diameter, shorter interspinous distance, and smaller angle *α* could help to predict the surgical difficulty of LaTME after NCRT in male LARC patients. We then constructed a nomogram predicting the surgical difficulty of LaTME following NCRT, which may assist surgeons when selecting surgical approaches preoperatively.

## Supplementary Information

Below is the link to the electronic supplementary material.

Supplementary file1 (DOC 38 KB)
