# Supplementary material for: MRI pelvimetry-based evaluation of surgical difficulty in laparoscopic total mesorectal excision after neoadjuvant chemoradiation for male rectal cancer
Source: Surg Today. 2021 Jan 9;51(7):1144–51. doi: 10.1007/s00595-020-02211-3 (PMC8215037; doi:10.1007/s00595-020-02211-3)
Supplement: Supplementary file 1 — Supplementary file1 (DOC 38 KB) [file 595_2020_2211_MOESM1_ESM.doc]

**Supplementary Table 1.** Definitions of MRI-based pelvimetric parameters

| **Measurements** | **Definitions** |
| --- | --- |
| Pelvic inlet | The distance between the sacral promontory and the upper edge of the symphysis pubis |
| Pubic tubercle height | The distance between the upper and the lower edge of the symphysis pubis |
| Pelvic outlet | The distance between the coccyx and the lower edge of the symphysis pubis |
| Pelvic depth | The distance between the center of pelvic inlet and coccyx |
| Sacral length | The distance between the sacral promontory and the tip of the coccyx |
| Sacral depth | The distance from the sacral length to the deepest point of the sacral hollow |
| Interspinous distance | The narrowest distance between the ischial spines at the maximal femur head level |
| The mesorectal fat area | The area subtracting the rectal area from the mesorectal area at the level of the tip of the ischial spines |
| Angle α | The angle between the upper edge of the pubic symphysis, promontory, and the middle of the S3 vertebra |
| Angle β | The angle between the line connecting the edge of the pubic symphysis and promontory, and the line connecting the lower edge of the pubic symphysis and coccyx |
| Angle γ | The angle between the line connecting the middle of the S3 vertebra and coccyx, and the lower border of the pubic symphysis |
| Angle δ | The angle between the line connecting the promontory and the middle of the S3 vertebra, and the line connecting the coccyx and the middle of the S3 vertebra |
